# Disordered eating among mid-age women: is quality of life impacted over time?

**DOI:** 10.1186/2050-2974-2-S1-O59

**Published:** 2014-11-24

**Authors:** A Kate Fairweather-Schmidt, Christina Lee, Tracey D Wade

**Affiliations:** 1 Flinders University, Adelaide, Australia; 2 School of Psychology, University of Queensland, Queensland, Australia

## Objective

This longitudinal study of midlife women has three aims: examine quality of life (QoL) among women with and without indicators of disordered eating (DE); compare the obtained QoL effect to a younger cohort in the same longitudinal study and investigate potential moderating effects of depression and social support on the relationship between DE and QoL.

## Method

We used self-report data from six waves of the Australian Longitudinal Study on Women’s Health over 14 years. A total of 12,338 women participating in the mid-age cohort (ageing from 45-50 to 59-64) provided self-report indications of DE at Surveys 1 and 2, and QoL (SF-36 component scales - mental [MCS] and physical [PCS]) at Surveys 2-6.

## Results

DE was reported by 10.25% of the women who also reported significantly poorer mental and physical QoL than those without DE; this effect was sustained over 12 years. Effect size differences for the midlife and younger women, between those with and without DE, showed a larger impact on physical QoL over time for the mid-age women. Midlife women with high levels of depressive symptoms (with and without DE) had the lowest initial mental QoL scores. Midlife women with high depression and DE had the greatest increase in mental QoL over time, but their mean score was still considerably lower than all other groups.

## Conclusions

This study underscores the significant effect of midlife DE on QoL, particularly when comorbid with depression, suggesting specific support options are needed for mid-age women.

This abstract was presented in the **Disordered Eating and Body Image** stream of the 2014 ANZAED Conference.

